# Clinical Lecture on Delirium Tremens

**Published:** 1842-09

**Authors:** George Budd

**Affiliations:** Prof. of Medicine


*Clinical Lecture on Delirium Tremens.* By George Budd, M. D., F. R. S,, Prof. of Medicine. Delivered at King's College Hospital, Nov. 20, 1841.—The case of Embleton is a fair specimen of the delirium brought on by excessive drinking. During the years I was physician to the Dreadnought, I had constantly cases of this kind under my care. The frequency of delirium tremens in sailors is very readily explained. When a ship returns from a long voyage, the crew are paid off. A man receives at once £40, £50, or £60, and is immediately laid hold of by the most despicable of human beings—the crimps, as they are called, or the agents of the keepers of the filthy lodging-houses in the neighborhood of Wapping. He is taken to one of these houses, and kept in a state of constant intoxication for a fortnight or three weeks. By this time his money is generally spent. His clothes are then pawned, and this supplies him with drink a day or two longer. When this last resource fails, when the system of robbery can be carried on no longer, the supply of drink is stopped, and the sailor is turned adrift to shift for himself. In a few days he is brought to the hospital in a state of delirium tremens.

I have known sailors, who had received as much as £70, reduced to complete destitution in the course of two or three weeks. During the time they are kept in a state of intoxication they eat nothing, often not so much as a morsel of bread, for days; they live, as they express it, on the liquor.

When they are brought to the hospital, the condition of all these men is very much alike. The face is flushed, the eyes are suffused, and there is often a peculiar wildness in their air and manner, even when there is no actual delirium. The tongue is pale, moist, and indented; and the hands as well as the tongue are very tremulous. Appetite is quite gone, and they have generally repugnance for food of every kind. In spite of this nervous agitation the pulse is steady and tranquil, and very seldom increased in quickness.

But what is characteristic of the disease, as characteristic as the trembling, is the loss of sleep. They all tell you that they have had no sleep for nights; and that the moment drowsiness comes over them, or even without this, at the approach of darkness only, they are haunted by visions of the most frightful kind; they see serpents about the bed, and are pursued by devils of every size and shape. The constancy of these horrid apparitions has furnished a name to the disease. The term, in fact, by which it is known to sailors, and a very appropriate one it is, is the 'horrors.'

The name has, no doubt, passed down through many generations of sailors. Smollet uses it in Roderick Random. Smollet, in early days, was a surgeon's mate in a man-of-war, and sketched Roderick from the life. Roderick, in relating his history, says, 'I awaked in the horrors, and found my imagination haunted by such dismal apparitions, that I was ready to despair.' This very properly happens to Roderick a day or two after he set sail. In delirium tremens from drinking, the patient generally gets more violent, and in every respect worse, a day, or two or three days, after he has left off drinking. Sailors are themselves aware that a glass of grog steadies them when in this condition.

In some cases, the men are more violently delirious: they are continually vociferating, and talking in the wildest and most incoherent manner; they are in a state of great agitation and tremor, and their body is bathed in sweat; yet even in these cases the pulse is generally calm and steady.

The character which the delirium assumes is that of hallucination. They see unreal objects, and these objects are almost always of a frightful kind. In some cases, but these are rare, the illusion extends to the sense of hearing. They hear the objects by which they are haunted, talking, and occasionally calling them by name.

But hallucination is more rare for the sense of hearing than for that of sight: probably because more vivid impressions are received through the eye than through the ear. Ghosts are more frequently seen than heard. Dr. Abercrombie thinks that dreams are chiefly occupied with objects of sight. In his work on the Intellectual Powers, he says, 'I am informed by a friend, who is a keen sportsman, that he often dreams of being on a shooting excursion; that he starts his game and points his gun, but never succeeds in firing it. It sometimes seems to miss fire; but in general there is something wrong in the lock, so that it cannot be moved.'

It was probably the poet's own experience that frequent and deep potations give birth to hallucinations of a frightful kind, that suggested to Burns the scene of the witches in Tam O'-Shanter. Tam, returning late after a drinking bout, passes by All Hallowe'en Church, in which he sees warlocks and witches in a dance, with Auld Nick for their piper. The objects with which the fancy of the poet has filled up the picture are certainly frightful enough:—

'Coffins stood round like open presses,
That shawed the dead in their last dresses;
And by some devilish cantrip slight,
Each in his cauld hand held a light,
By which heroic Tam was able
To note upon the haly table,—
A murderer's banes in gibbet-airns;
Twa span-lang, wee, unchristened bairns;
A thief new cuttit frae a rape
Wi' his last gasp his gab did gape;
* * * * * *
A garter which a babe had strangled;
A knife a father's throat had mangled,
* * * * * *
Wi' mair o' horrible and awfu',
Which e'en to name wad be unlawfu',

This description, though highly colored, is true to nature; but such hallucinations usually arise after prolonged excess, and not, as Burns makes it, after a single sitting.

Another character, very general in these cases, which seems allied to the frightful nature of the hallucinations, is despondency and a tendency to suicide. Even in slight cases I have known men suddenly burst into tears while I have been questioning them.

Well-marked cases of delirium tremens are easily recognised. Their characters are so striking, that, taken with the previous history of the patient, they can scarcely be mistaken; but this is not the case with the slighter degrees of the same affection. These are marked only by lowness of spirits, and inability to sleep; or by sleep which is interrupted by frightful dreams. It is a condition which is familiar to many by the name of the 'blue devils,' which, in the majority of cases certainly, must be ascribed to excess in drinking. Men of jovial habits—who take what is called a cheerful glass, and enjoy company and good cheer—are remarkably subject to the blue devils. They are low and desponding in the morning, and their nights are sleepless, or their sleep is broken by dismal dreams. In the language of sailors, they have the 'horrors.' It is the temperate man only whose spirits are uniformly good.

Embleton told us that the objects by which he was haunted, though continually shifting their shapes, were always of a blue color. I have often heard sailors make the same remark; and I am inclined to think that this is a very general character of such apparitions Müller, speaking of phantasms, says that they are generally divested of color.

I have said that in delirium tremens, the pulse, except after considerable muscular exertion, rarely exceeds its ordinary frequency. In cases in which it does, there is generally inflammation of some internal organ.

The organs most frequently inflamed are the stomach and the lungs.

It is not at all uncommon to find a man affected with the horrors, who, in addition to the ordinary symptoms—the delirium and the trembling—has frequent vomiting, a furred tongue, and a quick pulse. Such symptoms generally indicate inflammation of the stomach, and are of very bad augury; because, when they are present, the patient is unable to take solid nourishing food, which is a very important part of the treatment, and, indeed, frequently indispensable to recovery. In a case of this kind I have found the mucous membrane of the splenic extremity of the stomach reduced to a mere pulp.

The lungs are much less frequently inflamed than the stomach; but I have met with two instances of delirium tremens which proved fatal, apparently from the occurrence of pneumonia.

In none of the cases in which I have made an examination after death—in all, four or five—have I found any evidence of inflammation within the head, other than effusion of serum under the arachnoid. The symptoms do not depend on any inflammatory affection of the brain, but on exhaustion, consequent on over-excitement, and in part, perhaps, on the condition of the blood. We can hardly suppose that the blood should not become altered, and for some time remain so, by the continual admixture of spirits. The spirit undoubtedly exerts some degree of chemical influence.

Sailors, in whom I have chiefly observed delirium tremens, are men whose constitutions are not broken by habitual intemperance, but strong, robust men, most of them in the prime of life, who get drunk only while they are in port. From these circumstances the affection in them is somewhat different from what it is in the habitual drunkard; but the main characters and the indications of treatment are alike in both.

The chief remedy is opium; but it must be given in large doses, so as to procure sleep. In small doses, it does comparatively little good. When a man has been for days in a state of nervous agitation, and has had no rest for several successive nights, our object should be to procure sleep. I have often, as in the case of Embleton, seen a man in a state of raving delirium fall asleep under the influence of a large dose of opium, and, at the end of some hours, awake perfectly rational.

The plan into which I settled for the treatment of delirium tremens, such as I used to meet with in sailors, is to give one drachm of tincture of opium, every hour, for three or four successive hours, if the patient should not fall asleep before; and, as soon as he awakes, to give him a mutton chop, or some solid food.

I have already told you that these men, as long as their drinking bout lasts, eat nothing; and although, while the state of excitement continues, they have great seeming strength, and in some cases can be mastered with difficulty, yet, as soon as it subsides, they are always in a state of debility, and evidently require nourishing food. This is indicated, even while the delirium persists, by the condition of the tongue, which is almost always, in these cases, more or less indented; a condition you will generally find associated with states of debility.

Before they have slept they are unable to take food; their appetite is completely gone; a circumstance which must be attributed not to the state of the stomach merely, but to that of the whole nervous system; for the appetite generally returns when the system has been refreshed by sleep.

I consider this point—to give as soon as possible some solid food—a most essential one in the treatment; and have almost invariably found men get worse in cases where, from some mistake or neglect, it has been omitted.

I have said that opium should be given in large doses; but even these sometimes fail in procuring sleep. It is indeed astonishing how much of it can be taken by persons in this state of nervous excitement without sensible effect. In one instance in which I had left directions that a drachm of laudanum should be given every hour until the patient slept, ʒx. were given in the course of twenty-four hours, without producing the slightest sign of narcotism; and it was only after the last dose that the patient fell asleep.

A similar insensibility to the ordinary effects of opium is witnessed when it is given to relieve very severe pain or spasm. Doses, which in other circumstances would be dangerous, may be given not only with safety, but without apparent effect. But although large doses of opium may be given, and are even required, to relieve severe pain, or to control violent spasm, or to calm the nervous excitement of delirium tremens, you must not suppose thst its employment requires no caution in these cases. The rule generally laid down, and I believe it is a good one, is to discontinue the opium when it has produced contraction of the pupil. Different narcotic substances produce different effects on the pupil. Belladonna dilates it; the first effect of opium is to contract it. Contraction of the pupil shows, therefore, that the system is powerfully affected by the drug, and should caution us against its further employment.

When the opium acts favorably, when it procures a quiet sleep of several hours, the benefit resulting from it is remarkable: the delirium has ceased, the agitation has subsided, and, if the appetite have also returned, the patient is convalescent. But in some cases, opium, in however large doses we give it, fails in producing this effect. It calms the patient in some degree, and brings on a dosing state, which is often interrupted by startings, and from which the patient arises but little refreshed.

In many such cases, where opium had failed in composing the patient, I have seen admirable effects from wine or brandy, especially where the patient had left off drinking for several days. The delirium and nervous agitation seem to result partly from the sudden withdrawal of the stimulus. I have remarked that the men generally are much worse two or three days after they have left off drinking, and that they are aware that a glass of grog steadies them. But I would only give wine or spirit when opium had failed to procure sleep, and when the delirium had persisted some days after the patient had discontinued drinking.

Another important point in the treatment of delirium tremens is, that the patient should be removed as much as possible from all causes of excitement: his room should be quiet, and few persons should be allowed to visit him.

The last point that I wish to impress on you, is, the great importance of narrowly watching the patient. I have already stated to you that in this disease, even in its lighter shades, the patient is possessed by gloomy ideas, and has often a strong propensity to suicide. He should therefore be narrowly watched, and every instrument by which he can effect self-destruction should be placed out of his reach.

For want of these precautions we had a very tragic scene on board the Dreadnought, in the summer of 1838. Three men, Parrymore, Baily, and Forster, were brought into the hospital the same day in delirium tremens. They were treated in the usual manner, and I believe a straight-waistcoat was placed on each. The day after their admission, Parrymore was very much better, and gave me a clear account of many of the particulars of his case—as, of the voyage he had last made, the time he had been in port, and the money he had spent. All restraint was in consequence left off. The same evening he cut his throat with a clasped knife, which sailors generally carry about them. The surgeon, who was in immediate attendance, found him sitting up in bed, bleeding profusely, and still hacking away at his throat, to all appearance perfectly insensible to pain. The divided vessels were tied, and the hæmorrhage stopped; but he died at the end of a few days, from the secondary effects of the wound.

Baily also, the day after admission, gave me a clear account of many particulars of his case; and I considered him in no danger. The excitement and commotion in the ward consequent on the attempted suicide of Parrymore, again threw him into the most violent delirium, in which he continued for two days. On the fourth day after his admission, I found him very hoarse from vociferations, but tranquil and rational. The straight-waistcoat was taken off, and orders were given that he should be narrowly watched. The same evening, however, he eluded the vigilance of those about him, and threw himslf out of one of the port-holes into the Thames. A boat was immediately sent after him, and he was soon picked up, swimming lustily between the Dreadnought and the shore. When in the boat, he struggled violently to throw himself again into the river; and such was his muscular power, that it was with the greatest difficulty the two men in the boat could master him. They succeeded, however, in doing this, but Baily, apparently exhausted by his struggles, almost immediately expired. He was lifted into the Dreadnought a corpse.

We could not examine the body before a coroner's inquest had been held; and as this was not done for some days, and the weather was very hot (it was the middle of July), the body was too far advanced in decomposition to enable us to form a satisfactory opinion of the cause of his sudden death. I believe, however, that he died of pure exhaustion—the consequence of the powerful efforts he had just been making. He was one of the most muscular men I ever saw, and had no organic disease.

These cases, as you might expect, have made me very watchful over patients in delirium tremens. They should not be left a moment unguarded; and in hospitals where they cannot have a person in constant watch over them, I would always put on a straight-waistcoat, whenever I could do so without using violence or increasing the agitation of the patient. I make this exception, from a sense of the ill effects of excitement, and of the exhausting influence of great muscular exertion.

But delirium tremens, or at least a condition very similar to that brought on by excessive drinking, may be caused by any great shock to the nervous system, especially by surgical operations, or severe injuries.

When induced by this last cause, it is called traumatic delirium. But its characters are precisely like those I have before described:—nervous agitation, trembling, inability to sleep, and hallucinations, generally of a dismal kind.

The insensibility to pain (which I have before noticed in the case of Parrymore) in these cases is truly remarkable. Dupuytren, who has left a beautiful description of nervous delirium, says, that he has known patients in this state, with a comminuted fracture of the leg, slip off the bandages and walk about, using the broken limb without evincing the slightest pain; and others who had been operated on for hernia, introduce their finger into the wound, and amuse themselves with twisting about the gut, as if they were doing it on the dead body.

He also particularly remarks, as all writers on the subject do, the state of the pulse. He says, 'the most remarkable symptom, in the midst of this disturbance of the intellect, is the calm of the circulation, and the absence of every febrile symptom. You see a patient furious; his face covered with sweat; his eyes glistening; his cries heard at a distance: you believe him affected with the most intense arachnitis. Approach him: his pulse is calm and regular; and the state of his skin removes every suspicion of inflammation.'

But, in other circumstances, you will occasionally meet with a condition of the nervous system very similar to delirium tremens.

In the 19th volume of the Med. Chir. Trans. is a paper by Dr. Seymour, containing three cases of mania, successfully treated by morphia. They all occurred in women.

In the first case the woman became affected, after suffering severe distress, in consequence of the death of a relation who died in her presence. She was occupied with gloomy ideas, and a belief of having committed indescribable crimes. Her nights were sleepless, and there was continual watchfulness, and restlessness of the body. Her pulse was not weak, and her bodily health was unusually robust. She recovered in six weeks, taking a grain of acetate of morphia every night.

In the second case the history is obscure. The woman had been bled and blistered for what was conceived to be inflammation of the lungs. She was extremely violent; fancied that her children were murdered, and their spirits returned to torment them. Her sleep was broken. There was no fever. She recovered under the same treatment.

In the third case there was great previous affliction, and the mania came on after childbirth. The symptoms were nearly the same, and she recovered under the same treatment.

It is impossible not to perceive the analogy between these cases and those of delirium tremens. There is the same absence of fever, the same restlessness, the same want of sleep, and the occurrence of visions and dreams of the same character, in both.

All the instances I have mentioned agree in the absence of fever, and in the regularity of the functions of the body in the midst of the general nervous disorder; in the presence of gloomy ideas and frightful dreams.

It seems, then, probable that opium will be beneficial when ever there is a combination of these conditions. Dr. Seymousays,—'Morphia appears to be of more use in what is termed melancholia, where the mind is tortured by imaginary want, ruin, or crime, than where the derangement partakes of more brilliant ideas; as of superhuman knowledge, dexterity, or wisdom; or than in those cases where one single idea occupies and absorbs the mind.'

I have seen one or two instances in which a state very like that in the cases related by Dr. Seymour, resulted from excessive study.

One was in a man with whom I was well acquainted at Cambridge. He became insane after an attack of scarlatina in the summer of 1828. He had been reading excessively hard, and was much exhausted, when he was attacked with scarlatina on his way to the Lakes, to spend the summer vacation. He was laid up with scarlatina a fortnight; and soon after was placed in an asylum, where he remained two years. At the end of this time he was released, resumed his reading at Cambridge, and took a wrangler's degree in the year after that in which he would have taken his degree in the ordinary course.

The character of his insanity was like that of persons in delirium tremens. He fancied that he was constantly surrounded by devils; and even now, ten years since leaving the asylum, he is convinced of the reality of these apparitions.

My attention has lately been called to another case of the same kind. A man, like the former, became insane after hard reading; and his insanity was of the same character. He was placed in an asylum, where he continued some years. Since his release he has turned author, and the books he has witten display considerable talent.

I have little doubt that such cases are allied to delirium tremens—that the insanity is the cause of nervous exhaustion—and that it is best treated by opium, generous living, and quiet; means so efficacious in the various forms of delirium tremens. It would certainly afford a medical man a high gratification to cure a case of this kind—to restore an educated and gifted man to his family and himself; but there is very little satisfaction in curing a man of the delirium that results from drinking, because he is almost sure to get into the same state again. The only chance for him is to become a tee-totaler.

Experience of the 'horrors' has certainly very little influence in deterring sailors from a repetition of the excesses by which they are brought on. Embleton told us that he had had the 'horrors' twice before; and men have often confessed to me that they have had them as often as seven or eight times.

I am afraid, therefore, that Burns' parting advice, in Tam O'-Shanter, has been lost on the lovers of tippeny:—

'Whae'er this tale o' truth shall read,
Ilk man and mother's son tak heed;
When'er to drink you are inclined,
Or cutty sarks rin in your mind,
Think, ye may buy the joys owre dear;
Remember Tam O'Shanter's mare.'

